# Biomonitoring of lead in blood of children living in a former mining area in Lower Saxony, Germany

**DOI:** 10.1007/s11356-024-32719-x

**Published:** 2024-04-10

**Authors:** Sonja Strieker, Katja Radon, Felix Forster, Özlem Köseoglu Örnek, Laura Wengenroth, Walter Schmotz, Finn Sonnemann, Michael Hoopmann, Martin Hepp, Dennis Nowak, Tobias Weinmann, Stefan Rakete

**Affiliations:** 1 Institute and Clinic for Occupational, Social and Environmental Medicine, LMU University Hospital, LMU Munich, Ziemssenstr. 1, D-80336 Munich, Germany; 2 Department of Nursing Science, Universität Witten/Herdecke, Witten, Germany; 3 District of Goslar, Department of Construction & Environment—Soil Protection and Waste Monitoring, Goslar, Germany; 4 Public Health Agency of Lower Saxony, NLGA, Hanover, Germany; 5 District of Goslar, Department of Public Health Services, Goslar, Germany

**Keywords:** Lead, Spatial analyses, Environmental epidemiology, Mining

## Abstract

Environmental exposure to lead substantially decreased over the past decades. However, soil of former mining areas still contains high lead levels. We therefore performed a biomonitoring study among children living in two former mining communities in Lower Saxony, Germany. In these communities, soil contains lead levels of 1000 to 30,000 mg/kg. Overall, 75 children (6–10 years of age) attending the two primary schools of the study area took part in the study. Parents completed a short questionnaire on sociodemographics, and children provided capillary whole blood samples. We analysed lead using inductively coupled plasma tandem mass spectrometer. We compared the results to current German (20 μg/l for boys, 15 μg/l for girls) and US (35 μg/l) reference values. Potential associations between questionnaire information and lead results were tested using lead as continuous outcome and using lead dichotomized at the reference values. Finally, we analysed spatial patterns of elevated biomonitoring results. Of all children, 48% exceeded the German reference values for lead (5% expected) and 8% the US reference value (2.5% expected). Children 6–8 years of age were more likely to exceed German reference values (63%) than 9–10 year old children were (32%; *p*_Fisher_ = 0.01). No other questionnaire information was statistically significantly associated with biomonitoring results. Additionally, we did not find any indication of spatial clustering. In conclusion, we observed elevated blood lead levels in primary school children living in a former mining area. In the next step, exposure pathways need to be identified to implement effective public health measures.

**Supplementary Information:**

The online version contains supplementary material available at 10.1007/s11356-024-32719-x.

## Introduction

Over the last decades, exposure to lead decreased in many high- and middle-income countries due to the elimination of lead-containing gasoline, the ban of leaded pipes in water systems and other restrictions and agreements (Zhou et al. 2022). As a result, the concentration of lead in the human body declined as well in these countries (Hwang et al. 2019). However, soils of former mining and smelting areas are often contaminated with lead, and the contamination may persist even decades after the mining has ended (Liu et al. 2018). In areas with polluted soils, dust and food are important sources of exposure (World Health Organisation 2022).

In children, lead can impact the development of the central nervous system, potentially resulting in adverse effects on intelligence, behaviour (e.g., attention deficit hyperactivity disorder ADHD) and development (Olufemi et al. 2022). During pregnancy, maternal exposure to lead can cause developmental impairments of immature neuronal structures and can result in premature births and low birth weights (Caito and Aschner 2017). In addition, there is sufficient evidence for the carcinogenicity of lead in animals while evidence for humans is limited (International Agency for Research on Cancer 2020).

Even very low blood lead levels may harm. For example, around four points lower intelligence test results were found in children with blood lead levels between 24 and 100 μg/l (Lanphear et al. 2019). In a systematic review published in 2019, authors concluded that blood lead levels even below 30 μg/l are associated with symptoms of ADHD (He et al. 2019). In the absence of a known threshold, current reference values are generally based on the distribution of blood lead levels in a given population. For example, in 2021, the Center for Disease Control and Prevention in the USA lowered the reference value for lead in blood in children from 50 to 35 μg/l. This was done based on the 97.5 percentile for children aged 1–5 years participating in the National Health and Nutrition Examination Survey (NHANES) 2015/2016 and 2017/2018 (Centers for Disease Control and Prevention 2021a, b; Ruckart et al. 2021). In Germany, age- and sex-specific reference values are based on the 95th percentile of the general population. In 2019, the reference value, which was used in this study, was set to 15 μg/l for girls and 20 μg/l for boys between 3 and 10 years of age based on results of the German Environmental Health Survey 2014–2017 (Umweltbundesamt 2019; Vogel et al. 2021).

In certain parts of Lower Saxony (Northern Germany), mining and processing of ores containing lead, zinc, copper and silver ended in 1988. A recent analysis of soil samples taken in this area still showed very high metal concentrations especially of lead (up to 30,000 mg/kg) (Landkreis Goslar Fachbereich Bauen und Umwelt 2023). In an earlier biomonitoring study carried out in 1980, children (6–12 years of age) living in that region had median blood lead levels as high as 230 μg/l (Aurand and Hoffmeister 1980). Since that time, authorities in the study region have made extensive efforts to remediate soils and contaminated sites as well as to educate the public about the potential health effects (Schmotz et al. 2002; Daunert et al. 2010). The question arose whether the decreasing but still high environmental exposure levels still translate to a higher body burden. We therefore aimed at measuring the internal burden of lead in primary school children living in two adjacent communities in the contaminated region. Lead concentrations in these two communities are above 1000 mg/kg soil.

## Methods

### Study design

In autumn 2021, we carried out the cross-sectional study in primary school children living in the two adjacent communities Oker (district of Goslar, Lower Saxony, Germany) and Harlingerode (district of Bad Harzburg, Lower Saxony, Germany). The Ethics Committee at the Medical Faculty of the Ludwig Maximilians University of Munich approved the study (vote no. 20-0636). Legal guardians of all participants provided written informed consent. After completion of the study, we sent letters to the legal guardians informing them about the individual blood lead results.

### Study population

Schoolteachers invited all pupils (6–10 years of age) attending one of the two primary schools in the study region at the time of the study. According to the school records, at the time of the study, 163 and 221 pupils attended the primary schools in Harlingerode and Oker, respectively.

### Field work

Given that we carried out the fieldwork during restrictions of the SARS-CoV-2 pandemic, we did not have the possibility to present the study ourselves to parents or children. Therefore, the teachers informed the parents about the study in the regular parents’ evening and discussed the study with the children during school hours. To support the information campaign, we created information videos, posters and a study website. In addition, the study was announced through the local media. During the field phase, teachers distributed the study documents (study information, informed consent form and questionnaire) to their pupils.

### Questionnaires

A short questionnaire was offered in paper form (German), online (English, German and Turkish; Lime Survey GmbH, Germany) and as telephone interviews (German, English, Turkish and Arabic). The questionnaire included sociodemographic information only. This was done in order to keep the questionnaire simple, as we were concerned that the response might be affected given that we could not contact study participants ourselves nor were we able to conduct personal interviews due to pandemic restrictions. The legal guardians of the children answered the questionnaires. We used standardized questions and pre-tested their comprehensibility on four subjects.

### Blood sample collection and laboratory analyses

In November 2021, the study team visited the participating schools and collected capillary whole blood samples from the children’s fingertips. Sampling was done according to an adapted version of the University of Tennessee protocol (Centers for Disease Control and Prevention 2021a, b). In detail, a contact-activated lancet (blade 1.5 mm, penetration depth 1.6 mm; Sarstedt, Nürnbrecht, Germany) was used for pricking. The first drop of blood was discarded. Afterwards, we aimed to collect at least 250 μl of capillary blood into Microtainer® (BD, Franklin Lakes, NJ, US) tubes coated with K_2_-EDTA. Prior to the sampling, the blood collection tubes were tested for background concentrations. Samples were refrigerated and shipped on ice overnight to the laboratory, where they were stored at − 20 °C until analyses.

We analysed all samples on an inductively coupled plasma tandem mass spectrometer (ICP-MS/MS; 8900, Agilent Technologies, Waldbronn, Germany) equipped with a I-AS autosampler connected to an Ultra High Matrix Introduction (UHMI) interface. We tuned the instrument daily to achieve optimum sensitivity, oxide ratio and doubly charged ratio. Samples were analysed in HMI mode using a dilution factor of four. We ran all analyses in single quadrupole mode and used helium as collision gas to reduce polyatomic interferences. ^206+207+208^Pb was quantified using an external calibration. As internal standard, we used ^209^Bi.

All reagents used for analysis were of highest available grade. We obtained nitric acid for ultra-trace elemental analysis from Fisher Scientific (Optima™, Waltham, MA, USA). A water purification system (Direct-Q®, Merck, Darmstadt, Germany) provided ultrapure water (resistivity 18.2 Ω). We obtained elemental standards from Agilent Technologies (Waldbronn, Germany), lyophilized control materials for blood (lot 1299) from Recipe (ClinChek®, Munich, Germany) and prepared them according to the manufacturer’s instructions. For analyses, we thawed the samples on a roll mixer until they reached room temperature. We diluted an aliquot of 50 μl with 950 μl 0.5% (v/v) nitric acid containing 10 μg/l Bi as internal standard. Standards and control material were diluted in the same manner. The limit of quantification (LOQ) was 0.33 μg/l. All analyses were carried out in duplicate. For quality assurance, we ran control samples for blood at the beginning and the end of each run as well as every 20 samples. A recovery of 80 to 120% was considered as acceptable. Furthermore, the laboratory successfully participated in the German External Quality Assessment Scheme (G-EQUAS). Prior to the field work, the use of capillary blood for lead analysis and lead background levels of the capillary blood collection tubes was assessed (supplementary Material 1, supplementary Figure 1).

## Variable definition

Based on the questionnaire responses, we defined the following variables:

- Sex (“male” or “female”, as no participant reported their sex as “diverse”)
- Age in years (calculated by 2022 − year of birth)
- Level of education (“high” or “low” where “high” was defined as at least one parent with high school degree)
- Length of residence at current address (calculated by 2022 − year of moving-in)
- Exposure to second-hand smoking at home (yes/no)
- School attended by the child (“Oker” or “Harlingerode”)

We used lead in blood as continuous outcome variable. In addition, the absolute and relative frequency of samples above the reference value of 15 μg/l for girls and 20 μg/l for boys (Germany) (Umweltbundesamt 2019) and 35 μg/l (USA) (Centers for Disease Control and Prevention 2021a, b) were calculated.

### Statistical analyses

We performed all statistical analyses using the software packages R version 4.1.1 (38), SPSS (IBM SPP Statistics 26 26.0.0.1) and QGIS (QGIS 3.16).

First, we analysed the data descriptively. We present lead results as 10th percentile, median, 95th percentile, 98th percentile, maximum, arithmetic and geometric mean. This was done for the whole study population and for subgroups. Differences between groups were checked by Mann-Whitney *U* tests. In addition, we examined potential associations between metric variables (age in years; length of residence) and the biomonitoring results using the Spearman rank correlation. Variables associated with the blood level at *p* < 0.1 were included in a linear regression model. We also compared the absolute and relative frequency of samples above the reference values for subgroups using Fisher exact test.

In sensitivity analyses, we checked the robustness of our findings by excluding coagulated blood samples (*n* = 10) and blood samples with insufficient volume for double determination (*n* = 4). As for some families (*n* = 8) more than one child took part in the study, we also restricted the study population by randomly selecting one child per family.

### Spatial analyses

We investigated the geographical distribution of lead levels above the German reference value by analysing the distances between the children’s addresses by their biomonitoring results. If any spatial clustering was present, we would expect children with concordant results (both > or both ≤ the German reference value) living closer together than children with discordant results (one child > and the other child ≤ the German reference value). Therefore, we calculated a distance matrix using a geographic information system (GIS). We plotted and compared the distributions of distances for children with concordant and with discordant results.

Using the difference between the two distributions as an indicator for spatial clustering, we established upper and lower boundaries. For establishing the lower boundary (no clustering), we used the same distance matrix and randomly assigned (thus, simulated) biomonitoring results (lead above reference value or not) 500 times based on the found relative frequency of lead levels > reference value. For establishing the upper boundary (perfect clustering), we used the same distance matrix and simulated strongly clustered arrangements by declaring a starting child as > reference value and repeatedly doing so for the child that lived closest. We used four children as starting points (most northern/eastern/southern/western living child). We ended up with three strongly clustered arrangements, as two of those starting points were the same (i.e., two children living in the same house).

## Results

Overall, 89 parents completed the questionnaire. About half of the participating children were girls (*n* = 45); 40 children were living in families with higher level of education (56%). All children were born in Germany. The mean age of the study population was 8 years (range 6–10 years of age). The majority of the children (57%) had lived their whole life at their current place of residence (mean time at residency 7 years, range 1–10 years).

Prior to the field work, the blood collection tubes were tested for background concentrations, and the lead levels were below the limit of quantification (supplementary information). Furthermore, capillary blood samples were taken from a small number of volunteers and compared to venous blood samples (supplementary information, Figure S1). No significant differences were found between both sampling methods. During the main analysis, the recovery of lead in the quality control samples was between 98 and 118% (mean ± SD 105 ± 7%).

All of the 75 children who provided blood samples had lead levels above the limit of quantification. Almost half of the children (48%) exceeded the German reference values for lead while 8% had blood lead levels above the US reference value.

Stratifying the continuous blood lead results for sociodemographic subgroups (age; sex; parental education; domestic second-hand smoking; school; born at current place of residence) did not reveal any statistically significant differences between the groups (Table 1). Therefore, we did not conduct multiple linear regression analyses. Using the blood lead levels dichotomized for the German reference levels, 63% of the younger children (6–8 years) exceeded the reference levels compared to 32% among the older children (9–10 years; *p*_Fisher_ = 0.01). In addition, girls (59%) were non-statistically significantly more likely to exceed reference levels than boys were (36%; *p*_Fisher_ = 0.07). Using the US reference value as cut-off, higher vs. lower parental level of education and living in Oker vs. Harlingerode were associated with non-statistically significantly elevated blood lead levels (*p*_Fisher_ > 0.05).

**Table 1:** Blood lead results for the whole study population of children living in two former mining communities in Northern Germany and stratified for subgroups

	*N*	Percentile					Maximum	Mean		US reference level^d^		German reference level^e^	
		10	50	90	95	98		Arithmetic	Geometric	> 35 μg/l children 1–5 years		> 15 μg/l girls 3–17 years > 20 μg/l boys 3–10 years	
		μg/l								*n*	%	*n*	%
Total	75	8.9	16.9	31.9	37.0	53.3	65.8	18.8	16.9	6	8.0	36	48.0
Age (*p* = 0.11)^a^										*p*_Fisher_ = 0.67		*p*_Fisher_ = 0.01	
6–8 years	38	8.9	17.5	35.3	43.0	.	65.8	20.7	18.5	4	10.5	24	63.2
9–10 years	37	8.0	15.9	27.7	36.1	.	37.6	16.8	15.4	2	5.4	12	32.4
Sex (*p* = 0.24)^a^										*p*_Fisher_ = 0.42		*p*_Fisher_ = 0.07	
Female	39	8.0	16.1	27.1	37.6	.	65.8	17.8	15.9	2	5.1	23	59.0
Male	36	8.9	17.1	35.4	37.6	.	41.8	19.8	18.1	4	11.1	13	36.1
Parental level of education^b,c^ (*p* = 0.60)^a^										*p*_Fisher_ = 0.07		*p*_Fisher_ = 1.00	
Low	25	8.9	17.1	29.9	31.2	.	31.7	17.1	15.8	0	0.0	11	44.0
High	34	8.0	16.0	36.4	47.8	.	65.8	20.2	17.4	5	14.7	15	44.1
Domestic second-hand smoking (*p* = 0.57)^a^										*p*_Fisher_ = 1.00		*p*_Fisher_ = 0.70	
No	68	8.8	17.0	32.5	37.3	56.7	65.8	19.0	17.0	6	8.8	32	47.1
Yes	7	8.8	15.7	.	.	.	29.9	16.7	15.6	0	0	4	57.1
School (*p* = 0.35)^a, c^										*p*_Fisher_ = 0.06		*p*_Fisher_ = 0.61	
Harlingerode	53	8.4	16.6	27.3	33.1	35.8	35.9	17.4	16.2	2	3.8	24	45.3
Oker	22	8.9	17.5	40.6	62.2	.	65.8	22.0	18.8	4	18.2	12	54.5
Born at current place of residence (*p* = 0.43)^a^										*p*_Fisher_ = 0.51		*p*_Fisher_ = 0.16	
No	32	9.1	17.4	34.8	37.1	.	37.6	19.1	17.4	3	9.4	18	56.3
Yes	43	8.5	15.9	28.9	40.5	.	65.8	18.5	16.5	3	7.0	18	49.1

^a^Test for differences of central tendencies: Mann-Whitney *U* test

^b^High: at least one parent with high school degree

^c^Missing data: *n* = 16

^d^Centers for Disease Control and Prevention (2021a, b)

^e^Umweltbundesamt (2019)

### Spatial analyses

**Fig. 1:**
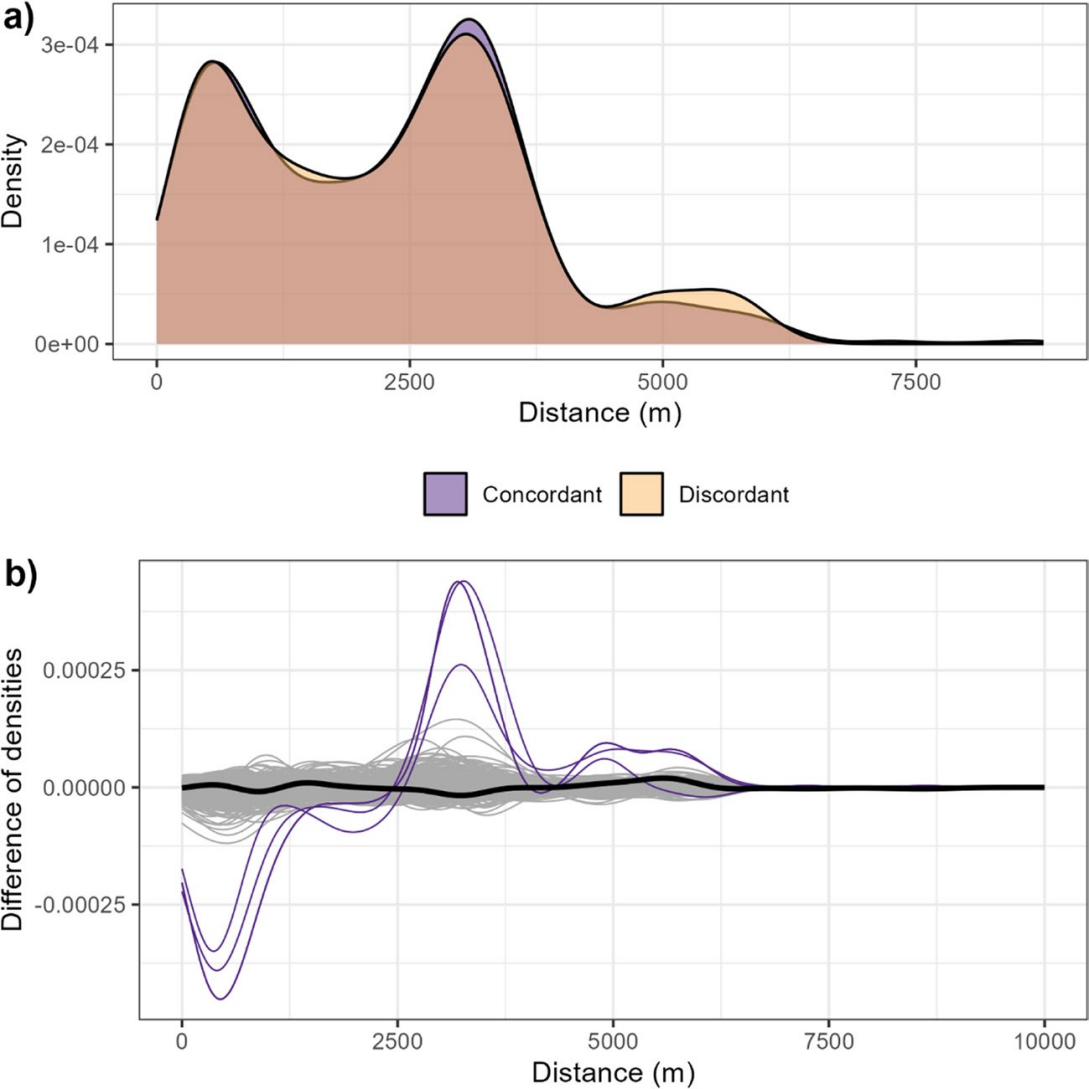
**a** Densities of distances between two children’s homes with concordant results (both children > or both ≤ reference value) and discordant results (one child > and the other one ≤ reference value). **b** Comparison of the difference of densities, comparing yellow curve minus blue curve from **a**, i.e., based on real data (bold black line), with 500 simulated density differences based on random assignment of biomonitoring results (thin grey lines) and density differences assuming strongly clustered arrangements of biomonitoring results (thin purple lines)

To further analyse any potential spatial clustering of blood lead levels, the distributions of distances between children’s addresses with concordant results (both children > or both ≤ the German reference value) and discordant results (one child > and the other one ≤ reference value) were compared. Results were similar (Fig. 1a). When compared with the lower and upper boundaries, the difference between the two distributions was similar to the lower boundary (i.e., no clustering; Fig. 1b).

## Discussion

In this cross-sectional study including biomonitoring results of 75 children of two former mining communities in Northern Germany, we found a higher burden of lead exposure compared to the general population. Almost half of the children exceeded the current German reference values for lead in whole blood (Umweltbundesamt 2019), and 8% were above the US reference values (Centers for Disease Control and Prevention 2021a, b). We could not identify groups of children with higher burden of lead exposure nor could we find any spatial clustering of lead levels. Therefore, the overall environmental background contamination might be considered the main source of exposure in these communities.

We recruited children via the two local public primary schools as they provide a relatively easy access to the population. In addition, given the compulsory education and the scarceness of private schools in the study area, our source population closely reflected the population of interest in the age range of 6–10 years. Assuming that all potential 384 families received the study information, response was 23%. Due to the SARS-CoV-2 pandemic, we were not able to contact the families directly, which might have limited our response. To address this issue, we have taken a number of measures to improve the response. However, compared to other studies, our willingness to participate was still relatively high. For example, the German National Cohort reached a response of 18% (Schipf et al. 2020). Nevertheless, the loss of about three quarter of sample reduced the representativeness of our study population, and our overall sample size was small limiting the statistical power of the study.

As participants were not aware of their exposure levels, it seems unlikely that participation was driven by biomonitoring results in the sense that, e.g., mainly children with higher levels of lead exposure were more eager to participate. However, considering the sociodemographic characteristics of our study population and comparing them to official statistics, we can assume that families with higher level of education and without migration background were more likely to participate.

Given the mining background in the area and the known exposure of the soil to lead, local preventive measures are in place. Those include information material on paper and online with recommendations on how to cultivate and consume fruits and vegetables in order to reduce exposure (Landkreis Goslar 2017). This material is so far only available in German and may thus result in less well-informed citizens with recent migration background. Furthermore, the explanations might be too complex for those with lower level of education or limited literacy. Unfortunately, we were not able to analyse food or hygiene behaviour in our study population given that we could not carry out personal interviews due to the pandemic restrictions in place at the time of the field work. Therefore, we decided to carry out a biomonitoring study as a first step to estimate internal exposure in the study population.

As in our study, capillary blood samples are increasingly used in epidemiologic studies given their in general greater acceptance compared to venous blood sampling (Centers for Disease Control and Prevention 2021a, b). We carefully validated the method in advance and piloted it in the field. Eight blood samples were partially coagulated, and four did not reach the minimum volume for double determination. Excluding these samples from the analyses did not change the conclusions of the study.

Median lead levels of participating children were less than 10% of the levels found 40 years ago following the trend also seen for the general German general population (Apel 2019). However, although exposure of children has been successfully reduced over time, for almost 50% of the population, blood lead levels were still higher compared to a recent representative population sample of children living in Germany (Umweltbundesamt 2019). Eight percent exceeded recent US reference values; however, in the US, only 2.5% of children aged 1–5 years exceeded the value of 35 μg/l. In addition, one has to take into account that also in the US, blood lead levels decrease by age groups (https://www.cdc.gosv/exposurereport/). Taking into account that blood lead levels below 30 μg/l may already adversely affect health results is to be taken seriously (He et al. 2019). We could not identify clear-cut risk factors for elevated blood lead results in our study, which might be due to the limited statistical power and, as discussed above, very limited questionnaire information. The trend for higher levels in younger age (not significant in our study though) is consistent with other studies and might be due to the hand-mouth behaviour and higher gastrointestinal uptake of younger children (Wani et al. 2015; World Health Organisation 2022). The finding that girls tended to be more likely to exceed German reference values than boys might partially due to the fact that reference levels in Germany still differ for boys and girls. The biomonitoring results of our study were comparable to recent blood lead data in children from another former mining area in Germany (arithmetic mean 13 μg/l) (Bertram et al. 2022).

Soil from all parts of the study area still exceeds the German precautionary value of 70 mg lead per kg soil (Landkreis Goslar 2017). Other similarly polluted regions show comparable lead levels in their population (Schoof et al. 2016; Rakete et al. 2022). Other studies, for example, in Kabwe (Zambia), which has lead levels in soil ranging from 139 to 62,142 mg/kg, children’s lead blood values were approximately 30 times as high as the ones measured in the present study (Bose-O'Reilly et al. 2018). The success of environmental education programmes in the study region might be an important reason for the observed differences (Daunert et al. 2010). In addition, differences in, e.g., time spent outside (Bertram et al. 2022), hygiene (e.g., availability of clean water to wash hands or food) (Morgan et al. 2017) and the dry climate increasing the inhalation of leaded dust (Laidlaw et al. 2005) might contribute.

The spatial analysis showed that the distribution of elevated lead values was close to how randomly distributed data would look like. In addition, we did not find differences in blood lead levels between the two schools. The generally elevated background exposure could be a reason for this lack of spatial distribution. One other potential source of lead exposure could be old leaded water pipes. However, we analysed 29 water samples of participating households and in only one household exposure slightly exceeded the current German limit value for lead (10 μg/l) in drinking water (data not shown). We therefore do not consider drinking water as a relevant source of exposure. The recent cross-sectional study in another mining area of Germany found a positive association between time spent in the garden with intense soil contact and the lead blood levels (Bertram et al. 2022). As mentioned above, we were not able to gather detailed information about potential pathways of lead exposure. However, this will be the focus of a future study in the region including more communities.

## Conclusions

Our findings revealed a substantial reduction in blood lead levels over time in children attending primary schools in a former mining area. However, lead levels were on average still elevated compared to their counterparts not living in mining areas. Future larger studies need to elucidate whether specific (behavioural) patterns can be identified as risk factors for our findings in order to develop improved preventive strategies for the region.

### Supplementary information

ESM 1 (DOCX 130 kb)
