# Supplementary material for: Biomonitoring of lead in blood of children living in a former mining area in Lower Saxony, Germany
Source: Environ Sci Pollut Res Int. 2024 Apr 10;31(20):29971–8. doi: 10.1007/s11356-024-32719-x (PMC11058760; doi:10.1007/s11356-024-32719-x)
Supplement: Supplementary file 1 — (DOCX 130 kb) [file 11356_2024_32719_MOESM1_ESM.docx]

**Supplementary Material 1**

Prior to the field work, the use of capillary blood for lead analysis was assessed. In detail, 19 volunteers provided both venous and capillary blood samples. From each participant, approximately 7 ml of venous blood were collected in Li-heparin tubes certified for trace metal analysis and subsequently stored in a -20 °C freezer until analysis. Capillary blood was sampled as described in the methods section. ICP-MS/MS analysis of these samples followed the same protocol as described in the methods section. The lead background levels of the capillary blood collection tubes were assessed by extracting 20 randomly selected tubes with 250 µl 0.5 % nitric acid for 2 h on a roll mixer. The extracts were treated as samples and analysed for lead as described in the methods section. Lead levels were below the limit of quantification

**Supplementary Figure 1**


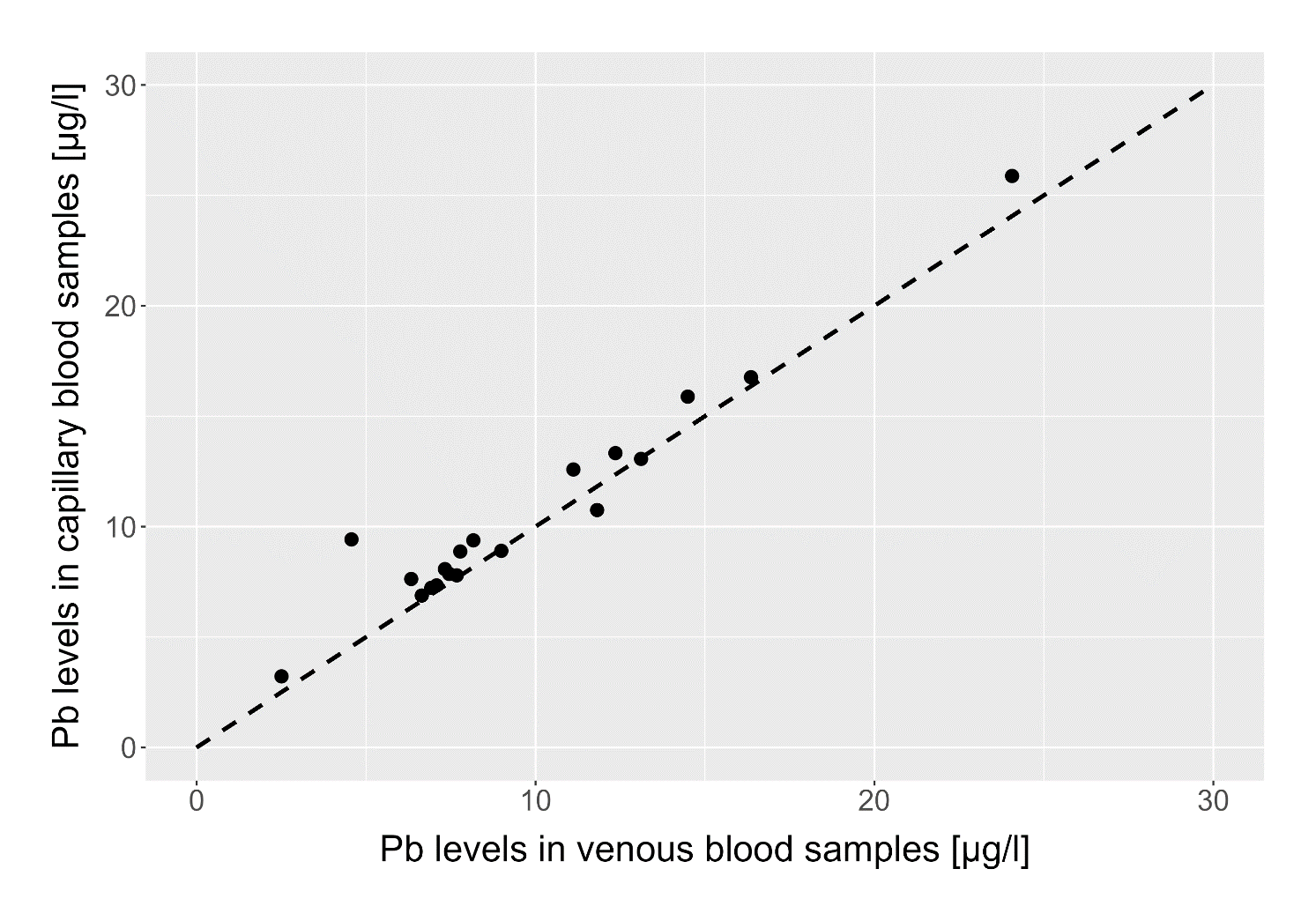


Scatter plot comparing lead concentration in capillary blood and venous blood samples from the pilot study (n=19). The dotted line is the identity line. Spearman-Rho correlation coefficient: R = 0.88, *p*<0.01.
